# Outcomes of Biliary Atresia in a Single Center in Jeddah, Saudi Arabia

**DOI:** 10.7759/cureus.27871

**Published:** 2022-08-11

**Authors:** Meshari A Alaifan, Sara H Simbawa, Tala A Fayoumi, Hotoun F Bokhari, Buthainah Al-Ghamdi

**Affiliations:** 1 Department of Pediatrics, Faculty of Medicine, King Abdulaziz University, Jeddah, SAU

**Keywords:** native liver survival, liver transplant, kasai procedure, jaundice, biliary atresia

## Abstract

Background

Biliary atresia (BA) is a rare but severe cause of obliterative cholangiopathy in neonates. Its incidence differs worldwide varying from 5/100,000 to 32/100,000 live births. The highest incidence is seen in Asia and the Pacific region. Diagnosing this disease is difficult in its early stages; thus, screening is necessary to avoid serious complications that can be minimized with early intervention during the first few months of life. Currently, although there are no medical treatments for BA, once the diagnosis is confirmed, the Kasai procedure may be a treatment option. The earlier the Kasai surgery is performed, the higher the success rate. Liver transplantation may be needed if the operation fails. This study aimed to determine the incidence of BA and the factors influencing the outcomes of the Kasai procedure at King Abdulaziz University Hospital, Jeddah, Saudi Arabia.

Methodology

This retrospective cohort study was conducted in the Pediatric Department at King Abdulaziz University Hospital, Jeddah from January 2019 to July 2019 and included consecutive patients with BA from 2010 to 2018.

Results

In total, 14 patients (57.1% female) were included in the study. The median age at the time of presentation was 90 (19-720) days, and the median age at the time of implementing the Kasai procedure was 90 (60-150) days. Eight patients underwent the Kasai procedure, and only one patient had a liver transplant.

Conclusions

Antenatal screening for BA tended to ensure early diagnosis and better outcomes. Delay in diagnosis and intervention is associated with increased morbidity and mortality.

## Introduction

Biliary atresia (BA) is a disease with an unknown cause, in which all or parts of the bile ducts are obstructed, leading to biliary blockage in newborns. Disease manifestation typically occurs within the ﬁrst months of life with jaundice, enlarged liver, and pale stool [1]. Prevalence rates depend on some risk factors, including country of residence, sex, race, maternal age, number of pregnancies, and birth weight. The prevalence of BA is higher in females than in males in the United States [2].

Diagnosing BA in the early stages is difficult, with ultrasound as the gold standard method for diagnosis. Ultrasound is recommended over other modalities such as liver biopsy because the histological findings of infantile cholestasis-causing disorders overlap [3,4].

Treatment of children with BA is mainly surgical and typically begins with an initial attempt to restore bile flow by Kasai portoenterostomy (KPE), which is considered to be an alleviating procedure, while liver transplantation (LT) can be a curable treatment option [5,6]. BA is the most common disease leading to liver transplants in the pediatric age group [6].

Previous studies have reported that the timing of KPE has a significant effect on the outcomes of BA. The two-year survival rate for children aged <60 days is 56.3%, that of patients aged between 61 and 90 years is 52.2%, and that of patients aged >90 days is 41.4% [7].

However, studies in Saudi Arabia focusing on the outcomes of BA and the factors affecting them are scarce. Thus, this study aimed to determine the incidence and outcomes of BA in Jeddah, Saudi Arabia.

## Materials and methods

This retrospective cohort study aimed to determine the incidence of BA and its influencing factors on the outcomes of the KPE at King Abdulaziz University Hospital (KAUH), Jeddah, Saudi Arabia. This study was conducted in the Pediatric Department of the hospital from January 2019 to July 2019. Our hospital is the first academic center in Saudi Arabia with a bed capacity of 1,002 and approximately 10,757 admissions yearly. This study was approved by the Research Ethics committee of KAUH (27-19).

The prevalence of patients with BA in the hospital from 2000 to 2018 was 54; however, due to the lack of documentation and scarcity, the sample size was reduced to 14 patients from 2010 to 2018.

The data were obtained from the hospital database using The International Classification of Diseases, Ninth Revision, Clinical Modification (ICD-9-CM) diagnosis code 751.61 and The International Classification of Diseases, Tenth Revision, Clinical Modification (ICD-10-CM) diagnosis code Q44.2, atresia of bile ducts. The collected variables included sex, nationality, birth date, gestational age of delivery, birth weight, current age, and clinical features at presentation (jaundice type and duration, pale stool, dark urine, hepatomegaly, splenomegaly, gastrointestinal (GI) bleeding, ascites, failure to thrive, other). Laboratory tests at presentation and last follow-up included liver enzymes (aspartate aminotransferase (AST), alanine aminotransferase (ALT), alkaline phosphatase (ALP), gamma-glutamyl transpeptidase (GGT)), bilirubin, vitamin D, mode of diagnosis (abdominal ultrasound, magnetic resonance cholangiopancreatography (MRCP), cholangiography, endoscopy, hepatobiliary (HIDA) scan, and liver biopsy), antenatal diagnosis, age at presentation, age at surgery, postsurgical complications (cholangitis, portal hypertension (HTN), hepatopulmonary syndrome, pulmonary hypertension, intrahepatic biliary cavities, and malignancies), regular follow-up, LT, living or deceased status, and age.

The inclusion criteria were all confirmed BA patients in the pediatric age group up to the age of 18 years according to the hospital’s policy by clinical, biochemical, and radiological findings.

The Statistical Package for the Social Sciences version 25 (IBM Corp., Armonk, NY, USA) was used for statistical analysis. Continuous variables are presented as mean and standard deviation, while categorical variables are presented as numbers and percentages. Paired t-test was used to evaluate the difference between the continuous and categorical variables. P-values of <0.05 were considered significant.

## Results

From 2010 to 2018, 14 children (six males, eight females) with BA were admitted to KAUH. All patients were born full-term with a median birth weight of 2.5 ± 0.5 kg. When categorizing patients according to their clinical presentation, 11 (78.6%) presented with jaundice, 10 (71.4%) with pale stool, four (28.6%) with dark urine, 10 (71.4%) with hepatomegaly, eight (57.1%) with splenomegaly, three (21.4%) with GI bleeding, seven (50%) with ascites, two (14.3%) with failure to thrive, and one (7.1%) with short stature. The median duration of jaundice was 73 (19-180) days, and the median age at the time of presentation was 90 (19-720) days.

Ultrasound was the most common modality used for initial diagnosis in 92.9% of the patients. Other modalities were used for further investigations and confirmation, such as MRCP (21.4%), cholangiography (28.6%), HIDA scan (14.3%), and liver biopsy (78.6%). None of the patients had any antenatal screening. Of the 14 patients, only eight underwent KPE, and only one patient had LT. The median age at the time of the KPE was 90 (60-150) days.

Follow-up complications included six (42.9%) cases of cholangitis, six (42.9%) cases of portal HTN, one (7.1%) case of pneumonia, one (7.1%) case of biliary cavitation, six (42.9%) cases of hepatomegaly, five (35.7%) cases of splenomegaly, six (42.9%) cases of ascites, three (28.6%) cases of sepsis, two (14.3%) cases of hepatopulmonary syndrome, one (7.1%) case of clubbing, one (14.3%) case of anemia, and one (7.1%) case of malignancy. Only nine patients came for regular follow-ups, and five were lost to follow-up. Three patients died at the following ages: 30, 120, and 150 days.

At the time of the study, 11 patients were alive, seven were two years old, and the remaining patients were one, three, six, and nine years old. We observed that eight patients who had other associated anomalies with BA, three patients had hernias (umbilical, inguinal, and abdominal), two patients had rickets, one patient had BA with splenic malformation (BASM) syndrome with polysplenia, one patient had coexistent Caroli disease, one patient had a congenital heart disease, one patient had esophageal atresia, one patient had systemic lupus erythematosus, one patient had gallbladder malformations, and one patient had a co-existent choledochal cyst. All laboratory findings are displayed in Table 1.

**Table 1:** The laboratory results of the patients pre-KPE and post-KPE. AST: aspartate aminotransferase; ALT: alanine aminotransferase; ALP: alkaline phosphatase; GGT: gamma-glutamyl transferase; KPE: Kasai portoenterostomy

	Mean results at presentation	Mean results at the last follow-up	P-value (significance at p > 0.05)
Patient laboratory results without intervention			
AST (U/L)	342.92	185.91	0.016
ALT (U/L)	252.57	121.61	0.004
ALP (U/L)	814.28	352.84	0.016
GGT (U/L)	475.57	139.30	0.018
Total bilirubin (µmol/L)	173.33	225.48	0.430
Direct bilirubin (µmol/L)	149.08	233.91	0.293
Vitamin D (nmol/L)	32.56	63.76	0.227
Post-KPE laboratory results			
AST (U/L)	177.08	185.91	0.807
ALT (U/L)	136.38	121.61	0.622
ALP (U/L)	452.30	352.84	0.198
GGT (U/L)	310.38	139.30	0.036
Total bilirubin (µmol/L)	148.91	225.48	0.222
Direct bilirubin (µmol/L)	122.73	233.91	0.128
Vitamin D (nmol/L)	32.70	63.76	0.393

## Discussion

BA is a rare congenital malformation of the bile ducts that results in obstructive jaundice, biliary cirrhosis, and liver failure [8,9]. The incidence of this disease differs worldwide varying from 5/100,000 to 32/100,000 live births and is the highest in Asia and the Pacific region [9]. However, even with the KPE and restoration of the bile flow, patients often have bad prognoses, resulting in the need to study the prognostic factors of this disease [8].

The comparative analysis of the post-KPE outcome aims to reduce the risk factors that affect the procedure. This time-sensitive success rate makes the infant’s age at the time of surgery crucial to optimize outcomes and decrease short- and long-term morbidity and mortality [10,11].

Outcomes of BA in our study varied due to different factors, such as early detection, age at the time of the procedure, and associated anomalies.

A French study conducted in 2005 with 63 patients reported that the median age at the time of operation was 69 days and ≥90 days in 12 patients. Twenty-year survival with the native liver was significantly higher in children who received operation before the age of 90 days [12]. An American study conducted in 2017 reported that the median age at the time of the KPE was 63 days [2]. In our study, the median age at the time of KPE was 90 days, which could have been a contributor to the poor outcomes for our patients. An Indian study conducted in 2017 revealed that patient outcomes were better when KPE was performed before the age of 60 days than when it was performed after 60 days [13]. Here, the success of KPE was affected by the late presentation to the hospital.

An American study that recruited 1,056 patients from nationwide hospitals from 1997 to 2012 reported that the incidence of BA was higher in females than in males (5.36 to 3.74 ratio) [2]. A single-center study in Japan recruited 119 patients from 1985 to 2004 and demonstrated that the incidence in females was higher than that in males (1.43 to 0.81 ratio) [14]. Another study conducted in Canada reported a 1.4:1 female-to-male ratio [6]. In our study, the percentages of females and males were 57.1% and 42.9%, respectively, indicating an insignificant difference.

In 2017, a multicenter retrospective study on commonly associated deformities of BA in China demonstrated that 4.94% of patients with BA had an associated anomaly. Heart defects were the most common, and other associated anomalies were pulmonary stenosis at 0.1%, intestinal atresia at 0.2%, situs inversus at 0.2%, and polysplenia at 0.3% [15]. Another study conducted in India in 2017 reported that 27.8% of patients had associated congenital anomalies, with 7.5% of patients having multiple anomalies. The results were as follows: 1.7% had cardiac anomalies, 2.5% had situs inversus, 3.3% had inguinal hernias, 10.7% had umbilical hernias, and 10.7% had polysplenia [13]. In this study, 69.2% of patients had associated anomalies, and the most common among them was hernias at 21.4%.

An increased incidence of hepatic malformations has been reported, especially splenic malformations (BASM syndrome), which is BA associated with a splenic abnormality (mainly polysplenia and less frequently asplenia). This syndrome, according to the literature, has poorer outcomes [8]. In 2007, a Canadian study revealed that 14% of patients with BA had BASM syndrome [6]. In our study, one patient had BASM with polysplenia and other complications, such as lymphoid neoplasm post-LT.

Infants with BA are typically born full-term with normal weight and a healthy general appearance. With increasing jaundice from the second day of life as the disease progresses, disease features emerge, such as pale stool, dark urine, hepatomegaly, and failure to thrive. In the later stages, splenomegaly may be found and indicates early cirrhosis [9]. In 2005, a retrospective study conducted in France recruited 63 patients and reported that 44 patients had splenomegaly before the diagnosis, 20 patients had GI bleeding before and after the KPE, and 17 patients had esophageal varices [16]. Here, 10 patients had hepatomegaly, eight had splenomegaly, seven had ascites, and three had GI bleeding, all at presentation. After the Kasai procedure, six patients still had hepatomegaly, five had splenomegaly, and six had ascites.

Early recognition of the disease is challenging because BA is a time-sensitive disease. Many common diseases, such as Alagille syndrome, alpha-1-antitrypsin deficiency, neonatal hepatitis, and other metabolic syndromes, should be excluded from the diagnosis of BA. Antenatal diagnosis can also occur at 20 weeks of gestation by observing cystic changes in the liver via ultrasonography.

Gallbladder abnormalities and the triangular cord sign are very accurate and commonly used ultrasound characteristics for diagnosing or ruling out BA [17]. Overall, the gold standard methods of diagnosis are cholangiography or liver biopsy [9]. We found that none of our patients underwent antenatal screening, which is strongly recommended.

A few weeks to months following the Kasai procedure, 30-60% of cases develop cholangitis, and at least 66% of cases develop portal HTN, even with a successful Kasai procedure [18-21]. A retrospective study from Australia reported similar results; 75% had ascended cholangitis, and 58.3% had portal HTN [22]. In 2017, a retrospective study conducted in India included the data of 121 infants, of whom three patients had portal HTN with GI bleeding, 10 patients had ascites, and 13 patients had increasing jaundice with yellow stools [13]. In our study, similar outcomes were observed: 42.9% of patients developed cholangitis, 42.9% of patients developed portal HTN, and 28.6% of patients developed sepsis.

If patients with BA develop biliary cirrhosis, the outcome is usually complicated, and if biliary cirrhosis recurs with ongoing cholangitis, the risk for hepatic malignancies increases. In 2001, a study from Japan reported three cases of BA complicated with hepatic tumors [23]. In our study, we found one patient in whom BA was complicated by lymphoma and who had a poor prognosis.

Despite successful treatment with the Kasai operation, BA remains an ongoing disease that requires careful management throughout life [24].

A limitation to our study is the nature of BA, which is a rare disease that is usually diagnosed late and is poorly documented in our hospital.

## Conclusions

Antenatal screening for biliary atresia is recommended to ensure early diagnosis and better outcomes. Delay in diagnosis and intervention is associated with increased morbidity and mortality. Multicenter prospective studies are needed to include biopsy review (size of the duct), medication protocol (steroid use), and TORCH screen, especially cytomegalovirus positivity, for better management of BA.
